# Anterior Cervical Huge Osteophyte Causing Dysphagia: A Case Report

**DOI:** 10.7759/cureus.37000

**Published:** 2023-04-01

**Authors:** Amin Gronfula, Thamer H Alsharif, Ahmed Deif, Ahmed A Fouda, Hesham Aboueleneein

**Affiliations:** 1 School of Medicine, Royal College of Surgeons in Ireland, Dublin, IRL; 2 Neurosurgery, Royal College of Surgeons in Ireland, Dublin, IRL; 3 Department of Surgery, Section of Neurosurgery, King Fahad Armed Forces Hospital, Jeddah, SAU

**Keywords:** trauma, quadriparesis, dysphagia, osteophyte, anterior cervical

## Abstract

The prevalence of anterior cervical osteophyte among elderly patients is high due to many causes such as trauma, degenerative changes, and diffuse idiopathic skeletal hyperostosis. Severe dysphagia is one of the main presenting symptoms for anterior cervical osteophytes. We describe a case of a patient with anterior cervical osteophyte with severe dysphagia and quadriparesis. The 83-year-old man presented to the emergency department following the incident of falling on his face. CT and X-ray were done in the emergency department, which showed huge anterior osteophytes at the level of C3-4 compressing the esophagus. The patient's consent was taken and shifted to the operation room and surgery was done. Anterior cervical osteophyte was removed, a discectomy was performed, and a peek cage and screws were inserted for fusion. In many cases of anterior cervical osteophyte, surgery is considered the ultimate treatment for patients to relieve symptoms, improve quality of life, and decrease mortality.

## Introduction

Elderly groups are more predisposed to have degenerative changes in the cervical spine, with a 75% or greater prevalence among people aged 65 or older with variable types of changes. Twenty percent to 30% of the elderly population was found to have an anterior cervical osteophyte. An anterior cervical osteophyte is usually found incidentally, as it is asymptomatic, but in rare cases, patients present with dyspnea, dysphonia, and dysphagia with a direct connection between hypertrophic spur size and symptoms. Osteophytes can be caused by trauma, diffuse idiopathic skeletal hyperostosis, ankylosing spondylitis, and degenerative changes [1-3]. Here, we present the case of an 83-year-old man presenting with dysphagia as a result of a large anterior osteophyte post-trauma, with a resolution of his symptoms following cervical osteophytectomy.

## Case presentation

An 83-year-old male, a known case of hypothyroidism and hypertension, came to the emergency department after falling and landing on his face. He presented with quadriparesis and severe dysphagia after the incident of falling. On examination, the patient was quadriplegic and wheelchair-bound; hyperreflexia was noticed on all limbs, and the Hoffman sign was positive. Regarding his dysphagia, he was not able to tolerate both liquid and solid diets.

**Figure 1:**
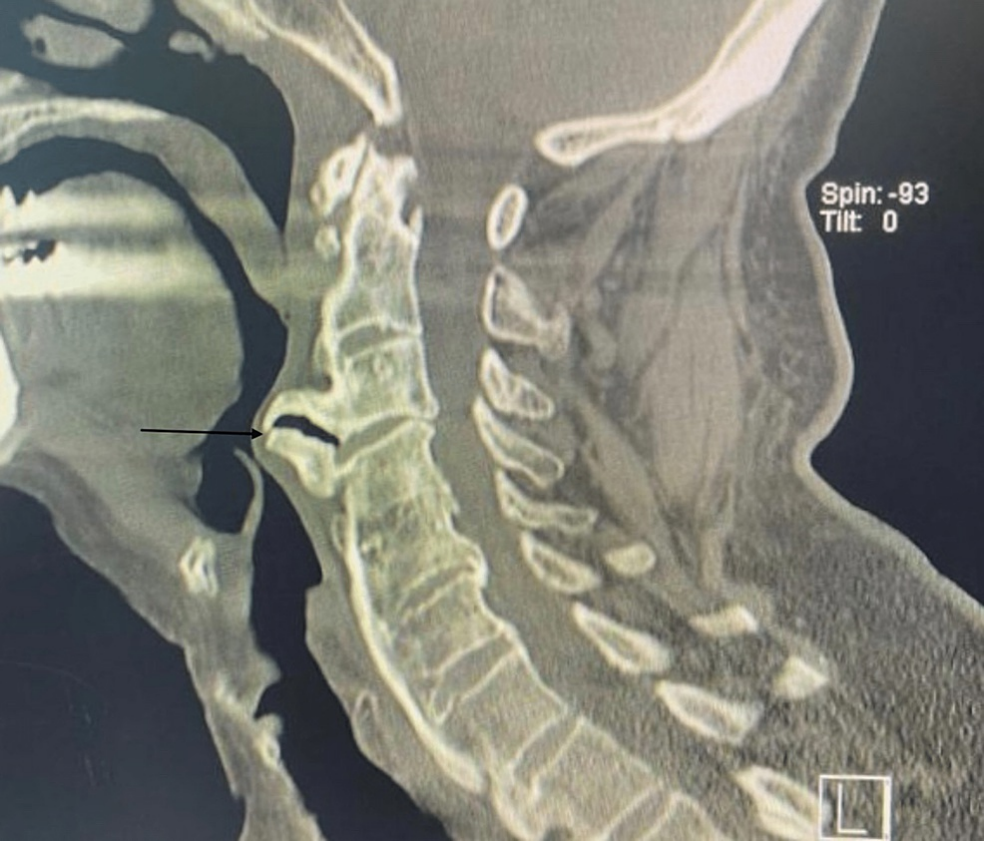
CT scan without contrast; sagittal view Huge anterior osteophytes were observed at the C3-4 level, compressing the esophagus (parrot beak sign)

**Figure 2:**
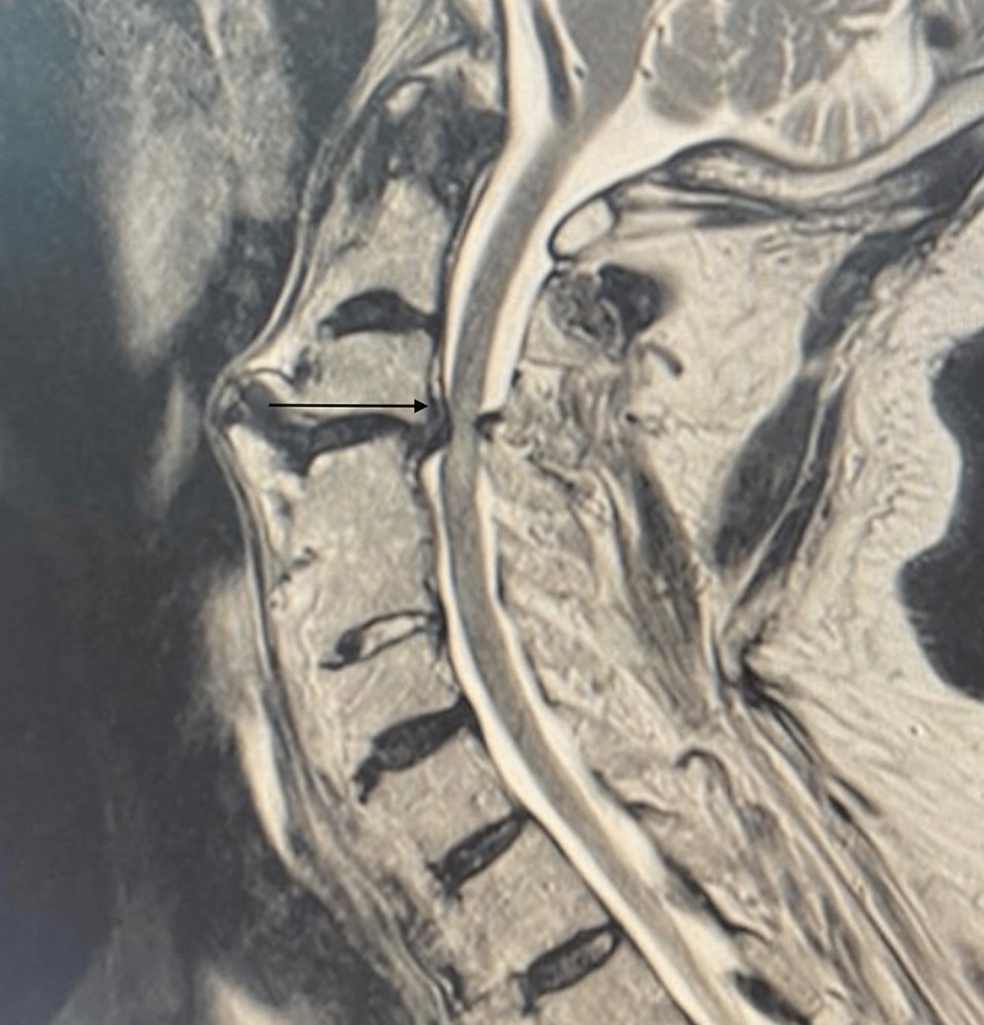
MRI cervical spine; sagittal view (T2) Showing a C3-4 cervical disc collapse compressing the spinal cord

**Figure 3:**
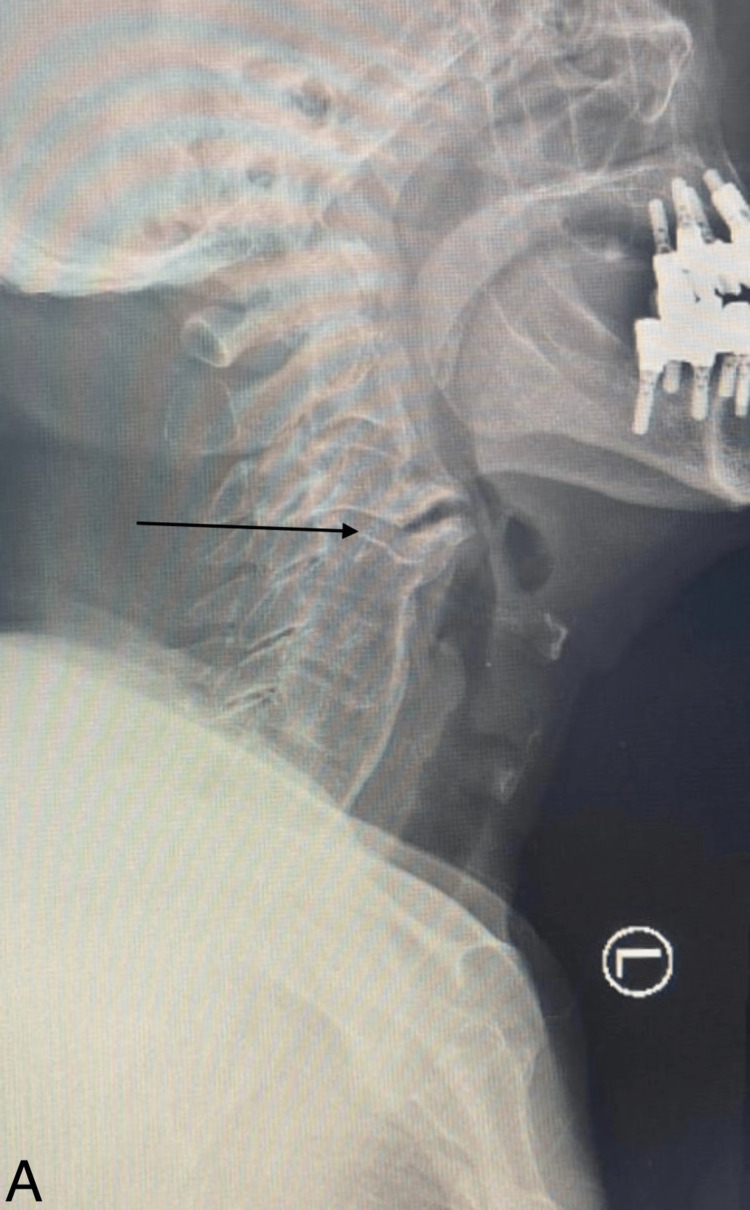
X-ray cervical spine; lateral view Showing anterior osteophytes at the C3-4 level

**Figure 4:**
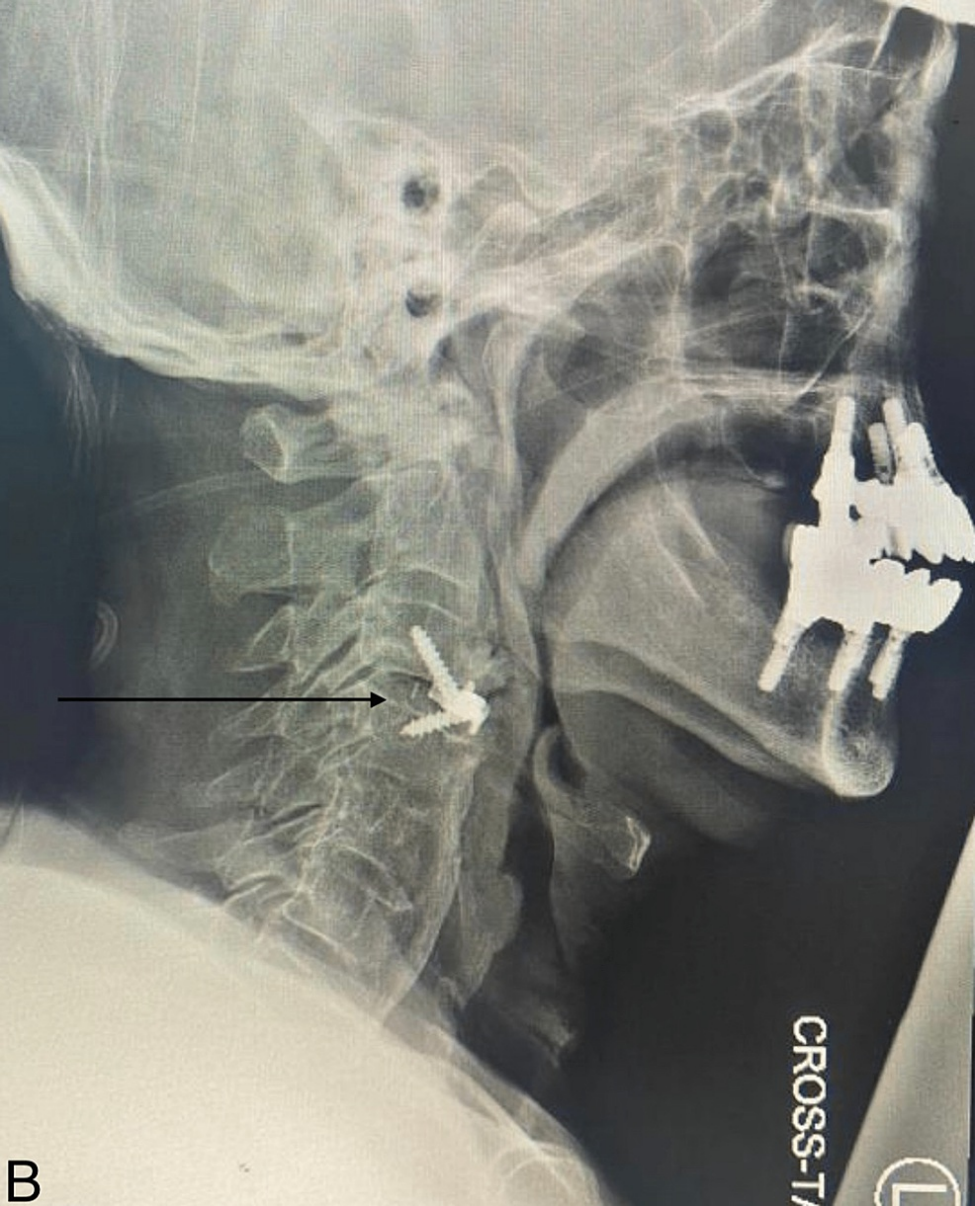
Postoperative X-ray cervical spine; lateral view Showing the removal of osteophytes with fixation of C3-4 by cage and screws

A computed tomography (CT) of the cervical spine showed a large anterior osteophyte spanning C3-C4. There was a suspicious-looking horizontal line at the superior aspect of the C5 vertebra. There was preserved vertebral height and shape of the rest of the vertebral bodies, with no evidence of subluxation. Diffuse degenerative changes are noted. Ossification of the posterior longitudinal ligament was noted at the C4 and C5 levels, causing the narrowing of the spinal canal. Calcification of the anterior longitudinal ligament was noted. A large anterior osteophyte was observed at C3-C4. CT showed disc herniation (Figure 1). MRI cervical spine sagittal view T2 showed C3-4 cervical disc collapse compressing the spinal cord at C3-4 (Figure 2). The anterior osteophyte was removed, followed by an anterior cervical discectomy and fusion, with the previous CT showing posterior ligament ossification. Postoperative X-ray showed the removal of osteophytes with fixation of the cervical spine (Figures 3-4).

The patient underwent surgery in a supine position, and pethidine preparation and anesthesia were done. The skin incision was guided by the superior capsular reconstruction (SCR) technique, identifying the C3-C4 level. The surgery started by opening the platysma, dissecting medially to the sternocleidomastoid muscle, and going down to the cervical spine. Identification of longus oculi and muscle stripping of longus oculi. Application of the Claud retractor and cuspal retractor and removal of the anterior osteophyte was done. Introduction of the microscope and then discectomy was done under microscope guidance and peek cage and screws were inserted for fusion. Closure of the platysma and skin was done, and the patient followed up with an MRI, physiotherapy, and rehabilitation.

## Discussion

Osteophytes can occur anywhere along the spine, but when they develop in the cervical area, they are usually asymptomatic. However, they can cause severe complications such as dysphagia. It was found by a study in the veteran population that 10.6% of patients who were evaluated for dysphagia had an anterior cervical osteophyte [4]. This might suggest that we could consider performing a CT scan for all patients with dysphagia after excluding all other common causes.

Patients suffering from dysphagia should undergo many investigations, such as manometry and a barium swallow test, to exclude common causes. An MRI is usually done for patients who have severe symptoms accompanied by dyspnea and dysphonia. MRI is done in patients diagnosed with anterior cervical osteophyte with severe symptoms pre-operatively to evaluate any stenosis that could be repaired at the time of surgery.

Observation and conservative treatment are the primary treatments for patients with dysphagia caused by a cervical osteophyte. A speech and language therapist would participate in the management to encourage swallowing exercises. Dietary modification and non-steroidal anti-inflammatory drugs are also used. Surgery would be the second option after the failure of conservative treatment [5].

Surgery is the mainstay of treatment in most cases, according to some surgeons. They believe cervical osteophytectomy should be considered in all cases of dysphagia caused by a cervical osteophyte to prevent progression to acute respiratory distress. Maiuri et al. supported this belief by submitting a case report of a patient who suffered from sudden respiratory distress requiring emergency tracheostomy in the background of chronic dysphagia caused by cervical osteophyte [6]. There are a couple of operative techniques, with resection of the osteophyte with or without spinal fusion being the most popular [7].

Osteophyte recurrence is not uncommon in osteophytectomy without spinal fusion, although it is considered to have a shorter operation time and causes fewer complications after the surgery [8].

## Conclusions

Physicians dealing with dysphagia of unknown cause among elderly patients should always suspect an anterior cervical osteophyte. A radiographic investigation along with a barium swallow test should be conducted on all patients with suspicion of an anterior cervical osteophyte to confirm the diagnosis. Ostephectomy is believed to be an adequate treatment for patients who fail conservative treatment to improve their quality of life and prevent severe complications such as respiratory distress.
